# Response to comments on “Spontaneous splenic rupture: A rare complication of concurrent malaria and dengue infections – A case report”

**DOI:** 10.1016/j.ijscr.2025.111459

**Published:** 2025-05-21

**Authors:** Abdirahman Omer Ali, Hassan Elmi Moumin, Ahmed Abdi Aw Egge, Mohamoud Hashi Abdi, Hamda Saed Olhayeh, Abdisalam Hassan Muse

**Affiliations:** a College of Health Sciences, School of Medicine and Surgery, Amoud University, Borama, Somalia; b School of Postgraduate Studies and Research, Amoud University, Amoud Valley, Borama 25263, Somalia; c Borama Regional Hospital, Surgery Department, Somalia; d Borama Regional Hospital, Radiology Department, Somalia

Dear Editor,

We are very grateful to the reviewer for their positive and encouraging feedback on our revised manuscript, “Spontaneous Splenic Rupture: A Rare Complication of Concurrent Malaria and Dengue Infections – A Case Report.” That work has been reported in line with the SCARE criteria [1].

We sincerely appreciate the reviewer's acknowledgment of the time and effort invested in addressing previous reviewer and editorial comments. We are pleased that our responses were considered careful and that the revisions have notably improved the manuscript's clarity and scientific rigor. It is particularly gratifying to hear that the expanded details regarding diagnostic methodology, therapeutic interventions, and especially the pathophysiological mechanisms underlying splenic rupture, including the discussion on dengue-associated splenic involvement and literature incorporation, have significantly strengthened the clinical value of the case. We also note the appreciation for our clarifications on resource-related limitations and adjustments to grammar and structure.

The following sections reiterate the detailed responses we provided to the earlier feedback (including that from Dehghani et al. [2]), which guided the revisions to the manuscript. We trust this provides a comprehensive overview of the changes made.

## 1 Diagnostic methodology: addressing ambiguity and providing specificity

We acknowledge the validity of concerns regarding the clarity and detail of our diagnostic methodology, as highlighted by both Dehghani et al. and the earlier reviewer feedback. Accurate differentiation between malaria and dengue is paramount given their overlapping clinical presentations, especially in resource-limited settings where comprehensive testing might be challenging.

Malaria Diagnosis: In this specific case, the diagnosis of Plasmodium falciparum malaria was established through microscopic examination of Giemsa-stained peripheral blood smears, which is widely recognized as the reference standard for species identification and quantification of parasitemia [3]. This method confirmed the presence of P. falciparum trophozoites. To corroborate this finding and provide a rapid result guiding initial management, a commercially available rapid diagnostic test (RDT) targeting HRP2 (Histidine-Rich Protein 2) specific for P. falciparum was also performed and yielded a positive result. While polymerase chain reaction (PCR) testing would have offered the highest diagnostic certainty and species confirmation, limitations in local resource availability precluded its routine use in this urgent clinical scenario.

Dengue Diagnosis: The diagnosis of dengue fever was primarily established through the detection of the non-structural protein 1 (NS1) antigen using a rapid immunochromatographic test. NS1 antigen detection demonstrates high sensitivity during the acute febrile phase (typically days 1–7) of infection [4], allowing for timely diagnosis. While initial serological testing for IgM and IgG antibodies was not performed concurrently due to the rapid positive NS1 result guiding immediate clinical decisions, subsequent retrospective testing using an ELISA method confirmed the presence of dengue-specific IgM antibodies, indicating a recent primary dengue infection. This sequential approach, necessitated partly by resource constraints and the need for rapid clinical action, ultimately confirmed the co-infection.

## 2 The therapeutic management and assessment of patient outcomes

The previous feedback rightly emphasized the importance of detailing the therapeutic regimen and patient outcomes. We hope the following provides the necessary clarity:

Antimicrobial Therapy: The patient was initially prescribed oral artemether-lumefantrine upon first presentation, following national guidelines which align with World Health Organization (WHO) recommendations for uncomplicated P. falciparum malaria [5]. However, due to severe vomiting and subsequent self-discharge, this was discontinued. Upon re-admission with hemodynamic instability and suspected complications, treatment was switched to intravenous artesunate (standard weight-based dosing) for severe malaria, again adhering to WHO guidelines.

Supportive Care: Supportive care for dengue fever and shock included aggressive intravenous fluid resuscitation (initially isotonic crystalloids like Normal Saline, titrated based on vital signs and clinical response), meticulous hematological monitoring (serial hemoglobin and platelet counts), and analgesic management (intravenous paracetamol for fever and pain). Antiemetics (metoclopramide) were used for nausea and vomiting. Blood transfusions (totaling 5 units of whole blood – 2 pre-operatively, 3 intra-operatively) were administered due to significant hemorrhage evidenced by low hemoglobin and hemoperitoneum.

Surgical Management of Splenic Rupture: The management of spontaneous splenic rupture (SSR) hinges on the patient's hemodynamic stability. Timely detection is essential for survival [4,5]. While conservative management (observation, embolization) can be considered in hemodynamically stable patients with contained ruptures or minimal bleeding [6], surgical intervention is mandatory in cases of persistent hemorrhage or hemodynamic instability, as seen in our patient (hypotension, tachycardia, evidence of massive hemoperitoneum on CT). The decision for an emergency laparotomy and total splenectomy, rather than spleen-preserving techniques (e.g., splenorrhaphy, partial splenectomy), was based on the intraoperative findings of multiple deep lacerations on the medial surface of an already enlarged and friable spleen, coupled with significant ongoing hemorrhage (estimated 4 l intra-abdominally) and the patient's instability. Emergency splenectomy remains the definitive treatment in such life-threatening scenarios to achieve hemostasis rapidly [4,5,7,8].

Post-Splenectomy Care & Outcome: Postoperative care focused on hemodynamic monitoring, pain control, and infection prevention. The importance of vaccination against encapsulated pathogens is paramount following splenectomy. Our patient received vaccinations against *Streptococcus pneumoniae* (Pneumococcal conjugate vaccine, PCV13, followed by Pneumococcal polysaccharide vaccine, PPSV23, schedule), *Haemophilus influenzae* type b (Hib), and *Neisseria meningitidis* (Meningococcal conjugate vaccine, MenACWY) approximately 2–4 weeks post-discharge, consistent with established guidelines to mitigate the risk of overwhelming post-splenectomy infection (OPSI) [9,10]. While OPSI risk persists long-term (incidence estimated at 4–5 % in some series, occurring potentially years post-surgery [8,11]), timely vaccination significantly reduces this risk. The patient's postoperative course was unremarkable; drains were removed on day 5, and he was discharged on day 7 in a stable condition. He received thorough education on infection risks, the need for prompt medical attention for febrile illnesses, and adherence to lifelong prophylactic measures (including potential antibiotic prophylaxis depending on local guidelines and risk factors) and booster vaccinations.

## 3 Pathophysiological mechanisms underlying splenic rupture

We concurred with Dehghani et al. and the previous reviewer that a more in-depth discussion of the underlying pathophysiological mechanisms contributing to splenic rupture in the context of malaria-dengue co-infection was warranted, and we are pleased this was well-received. While spontaneous splenic rupture is a recognized, although rare, complication of malaria, it is less frequently associated with dengue fever.

Malaria: Malaria-associated splenic rupture is primarily attributed to a combination of factors, including splenic congestion secondary to the sequestration of infected and uninfected erythrocytes, significant hyperplasia of reticuloendothelial cells (macrophages and lymphoid tissue) in response to the parasitic infection, and endothelial dysfunction, ultimately leading to microvascular occlusion and potentially splenic infarction [[12], [13], [14]]. This confluence of factors dramatically increases intrasplenic pressure and renders the enlarged, congested organ structurally fragile and susceptible to rupture.

Dengue: As the reviewer noted positively, the mechanisms for dengue-induced splenic injury and subsequent rupture, while less common than in malaria, are multifaceted and extend beyond simple thrombocytopenia. Proposed mechanisms involve:

Immune-mediated processes: These are complex and may include a cytokine storm with elevated levels of pro-inflammatory mediators (like TNF-α, IL-6) contributing to systemic vascular leakage, which also affects the spleen, causing edema and stretching of the capsule. Furthermore, T-cell mediated responses targeting virus-infected cells within the spleen, and potentially immune complex deposition activating the complement system, could contribute to inflammation and parenchymal damage [15,16].

Increased Vascular Permeability and Endothelial Damage: A hallmark of dengue infection, increased vascular permeability leads to fluid extravasation into splenic tissue. Direct viral invasion of splenic endothelial cells has also been postulated, potentially causing direct damage and contributing to fragility [17].

Coagulopathy and Hepatic Involvement: While thrombocytopenia is common in dengue, coagulation factor deficiencies, potentially linked to dengue-associated liver dysfunction, may also play a role [17]. This coagulopathy could contribute to the formation of subcapsular hematomas or exacerbate bleeding following minor parenchymal tears within the spleen, increasing the risk of overt rupture.

Co-infection Synergy: The synergistic effect of these overlapping and potentially compounding mechanisms in co-infected individuals likely amplified the risk in our patient. The intense splenic congestion and cellular sequestration driven by P. falciparum malaria, combined with the dengue-induced cytokine-mediated inflammation, vascular leakage, potential direct endothelial damage, and coagulopathy, probably resulted in a far more pronounced splenomegaly and critical structural weakening than either infection might typically cause alone. This heightened fragility, stemming from both mechanical stress (congestion) and inflammatory/vascular compromise (dengue/malaria immune responses), likely precipitated the spontaneous rupture observed. Further investigation is needed to fully elucidate this complex interplay.

## 4 Conclusion

Once again, we sincerely thank the reviewer for their positive assessment and for recognizing the improvements made to the manuscript. We also reiterate our gratitude to Dehghani et al. for their insightful earlier critique. We are glad that our elaborated explanations regarding the diagnostic process, therapeutic decisions, and potential pathophysiological mechanisms have successfully addressed the previous concerns and enhanced the manuscript's clarity and detail. This case indeed underscores the critical need for heightened clinical vigilance in malaria- and dengue-endemic regions and highlights the importance of considering SSR in patients with acute abdominal pain and concurrent infections. We remain committed to providing comprehensive details in our future publications. Future research should focus on elucidating the precise pathophysiological synergy in co-infections and developing optimized diagnostic and management strategies for this rare but life-threatening complication.

## Author contribution

All authors contributed equally to the writing, review, and editing of this response.

## Consent

Written informed consent was obtained from the patient for publication and any accompanying images. A copy of the written consent is available for review by the Editor-in-Chief of this journal on request.

## Ethical approval

The study protocol, case investigation, and consent form were thoroughly examined by the institutional review board of the College of Health Sciences at Amoud University. They granted approval for the study, along with the Ministry of Health and Borama Regional Hospital in Awdal Region, Somaliland (BRHH-160/2024). Prior to participation, written informed consent was obtained from every individual involved.

## Guarantor

All the authors of this paper accept full responsibility for the work and/or the conduct of the study, had access to the data, and controlled the decision to publish.

## Research registration number

Name of the registry: Not applicable.

Unique Identifying number or registration ID: Not applicable.

Hyperlink to your specific registration: Not applicable.

## Funding

This study received no external funding.

## Conflict of interest statement

The authors declare no conflicts of interest.
